# Crush of Plasmatic Flesh: Dual Reproduction of Characters and Abjection in Manga *Dragon Ball*

**DOI:** 10.12688/f1000research.159864.1

**Published:** 2025-02-26

**Authors:** Kohki Watabe

**Affiliations:** 1 Institute of Humanities and Social Sciences, University of Tsukuba, Tsukuba, Ibaraki, 3058577, Japan

**Keywords:** Dragon Ball, Manga, Toriyama Akira, Metamorphose, Plasmaticity, Kyara (Proto Character)

## Abstract

**Background:**

This paper analyzes the mechanism of the proliferation and disappearance of graphical characters in the manga *Dragon Ball*, a globally successful media franchise. *Dragon Ball* was serialized by Japanese manga artist, Toriyama Akira (1955–2024), in *Weekly Shonen Jump* magazine between 1984 and 1995. The manga, published in 42 volumes, was animated for TV from 1986 to 1996 as *Dragon Ball* and *Dragon Ball Z.*

**Methods:**

In support of the arguments on animation, such as Susan Napier’s metamorphosis, Sergei Eisenstein’s plasmaticity, and theories on characters in anime and manga studies, this paper analyses visual properties of characters in Toriyama’s *Drgaon Ball*, with a special focus on the graphical and biological reproduction.

**Results:**

This paper found that characters in *Dragon Ball* operate as a vessel for the shape-shifting fluidity of ink lines. The free kinetic movement of lines results in the graphical proliferation of characters and reproduction devoid of sexual expression. This is a version of the “abjection” — a reaction of the subject to reject familiar things as something horrific — that Napier argued regarding metamorphosis in turn-of-the-century Japanese anime. In *Dragon Ball*, muscular men’s battles hide the potentially grotesque graphic/biological proliferation of the characters. What stops this medium-specific proliferation of images is light as the lack of ink, a media-aesthetic element inherent to manga and anime. It is not flesh-and-blood combat but energy waves that inflict death on the villains who resurrect even from a single cell, just as lines drawn in ink multiply endlessly.

**Conclusions:**

These findings offer rich insights into the history of visual popular media in post-war Japan. The glow of the energy waves expressed by the lack of ink echoes the repeated motif of mass destruction in the history of Japanese anime and manga, which reminds us of the extinction of lives in the atomic bomb explosions.

## 1. Introduction

Several initial volumes of the *Dragon Ball* manga series are adventure stories in a multispecies world with an oriental and sci-fi touch. Modeled after *Journey to the West* — a 16 ^th^-century Chinese novel depicting the legendary Buddhist monk’s pilgrimage to Central Asia and India — the manga series follows Son Goku, a monkey-tailed boy. The wild boy who grew up in the countryside meets Bulma, a city girl who embodies the sci-fi elements of *Dragon Ball.* With a father who is an inventor and president of Capsule Corporation, which develops and manufactures Hoi-Poi capsules that allow various machines to be carried around in the form of small capsules, Bulma shows off high technologies to the boy who has never even met a girl before. They travel the world in search of the seven Dragon Balls, which are likely to grant a wish of the possessor. This idyllic adventure storyline in a world filled with demi-humans, aliens, and animal people who understand human languages gained relatively little popularity; however, in response to readers’ expectations, the genre was gradually switched to battle manga, which emphasized combat with evil antagonists ( Watanabe, 1995, 262-264). The battle element and muscular characters, rather than the early adventures, supported the worldwide popularity of *Dragon Ball.*

The question of where the source of *Dragon Ball*’s global popularity lies demands precise discussion. The change of genre from adventure to battle can explain that the main attraction of this work is the fight between muscular men. For example, Bounthavy Suvilay (2018, 250-267) points out that *Dragon Ball* expresses unique masculinity through the influence of the sports manga narrative tropes and kung fu stars such as Jackie Chan and Bruce Lee, although not intact by comical elements. Since Bandai, which manages the copyrights, has developed an abundance of figures of muscular soldiers ^1^ and fighting video games, and several male athletes and rappers ^2^ who sell their masculinity express themselves as fans, masculinity seems to play a key role in appealing to the fans to a certain extent. However, the story of *Dragon Ball* is not simply a battle of bloodthirsty men. For example, Piccolo and Vegeta, who were enemies in the first half of the story, awaken to fatherhood, and Gohan, the strong son of the protagonist, hates fighting and tries to avoid it ( Oya, 2023, 257-269). Hence, what place do the fights between muscle-bound men, which are a significant part of the work, have in the overall world of *Dragon Ball*?

**Figure 1.**
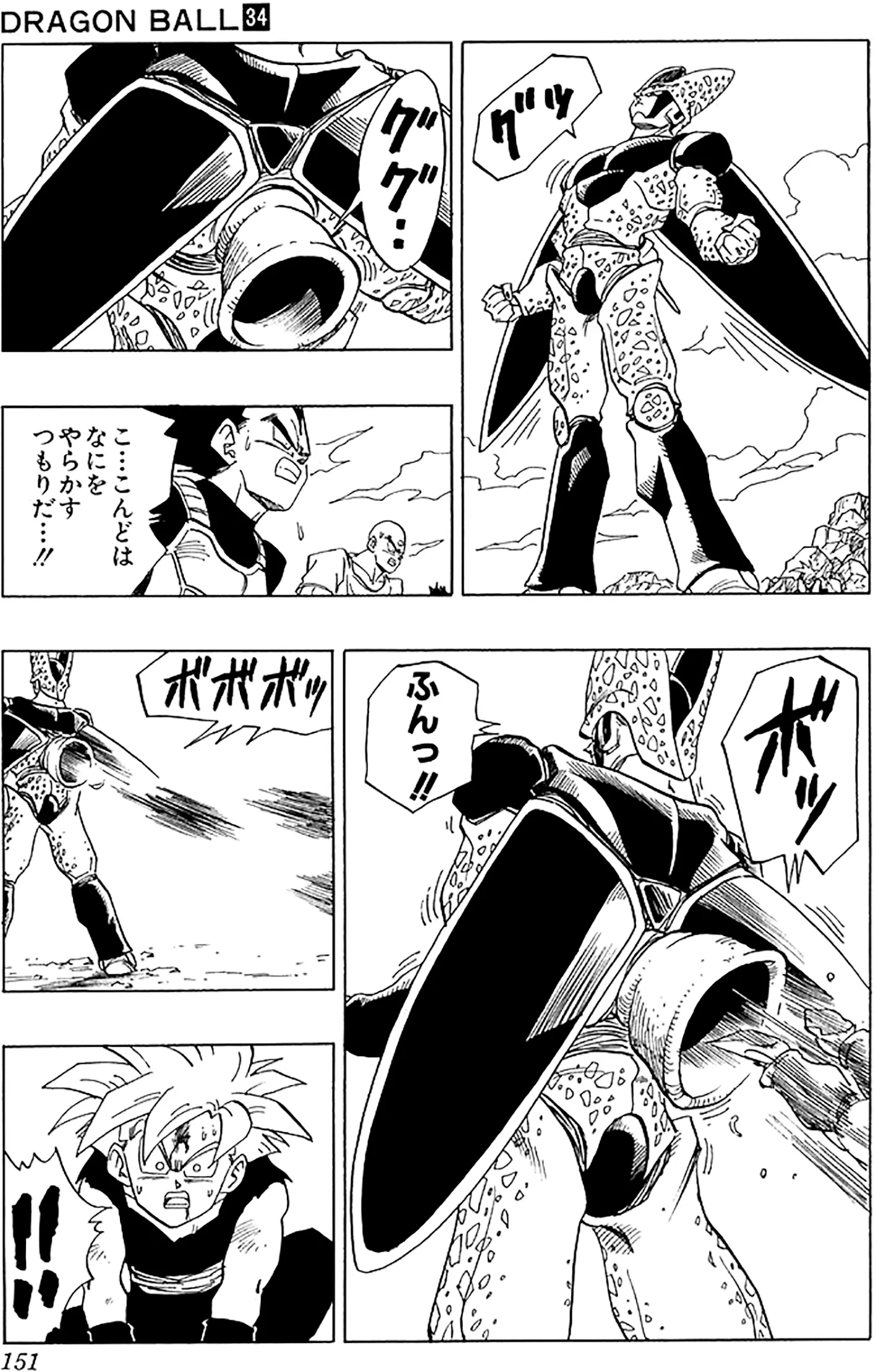
Cell’s “Delivery” (Chapter 406).

**Figure 2.**
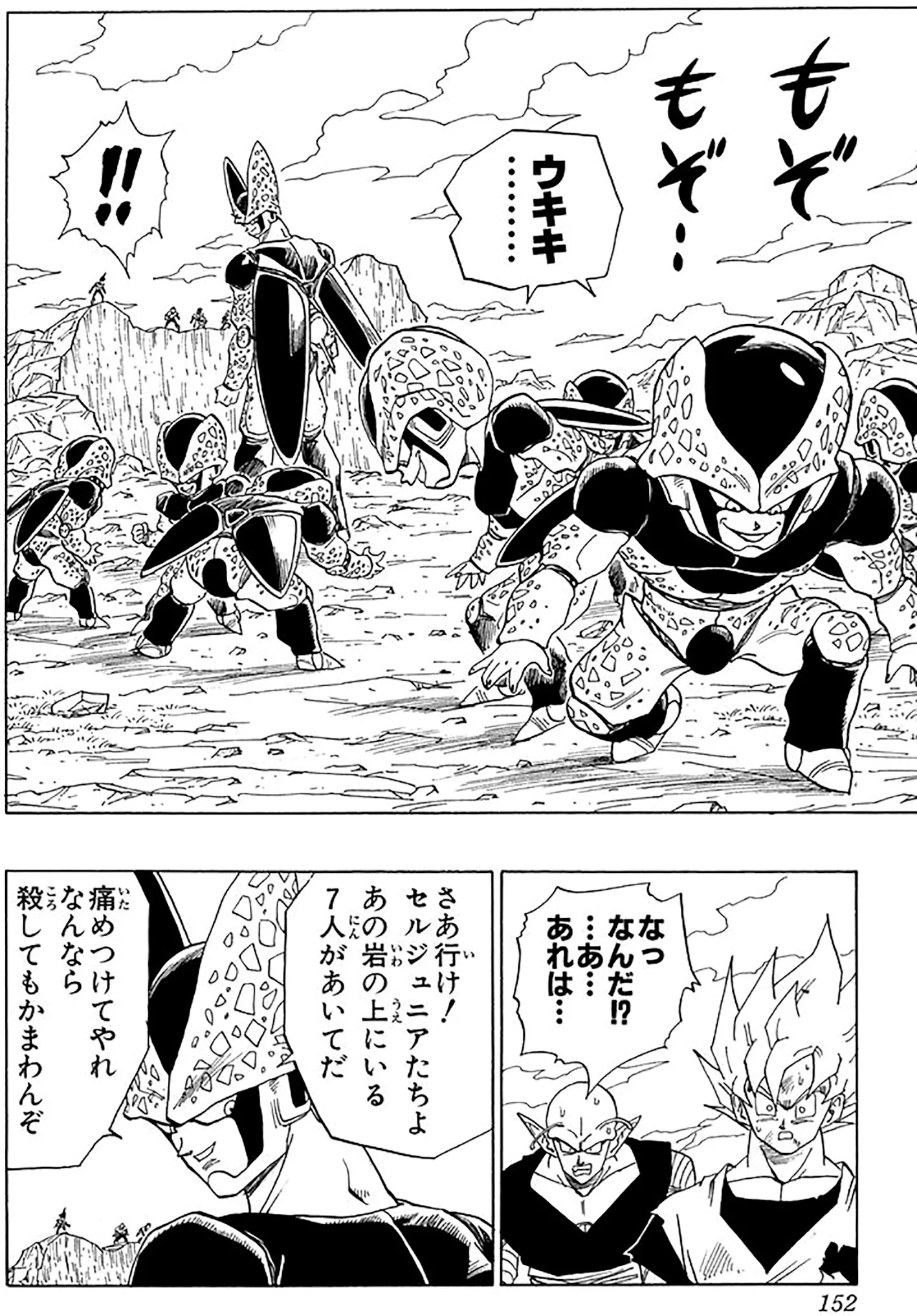
Cell and Cell Juniors (Chapter 406).

To answer this question, this paper analyzes the images in the 42 volumes and examines the complex potential of this text. By focusing on the visual representation of the manga, rather than on the narrative, this paper reveals that biological reproduction and the fear of it are embedded in the muscular battles in *Dragon Ball.* Cell, one of the major villains of the manga series, gives birth to seven organisms, called Cell Juniors, to match the number of warriors on the side of the protagonists ( Figures 1 and 2) (Toriyama, Ch. 407). ^3^ Cell is an artificially created life form whose sex is not explicitly stated and does not seem to reproduce sexually through the male-female binary. Cell was created to defeat Goku; therefore, the manga foregrounds the battles with the men on the protagonist’s side. Thus, if viewed emphatically, Cell’s metaphorical “delivery” scene is discordant with its hungry-for-a-fight masculinity, an undeniable attraction of *Dragon Ball.* By analyzing the graphic elements whose meanings are irreducible to the story, as in the case of Cell, this paper finds that biological reproduction challenging the appeal of fighting muscular men appears inconspicuously everywhere.

## 2. Methods: Metamorphose, Plasmaticity, and Sexuality in Anime and Manga Studies

This study mobilizes discussions in both manga and anime studies to analyze Toriyama’s *Dragon Ball.* The two media have different specificities: manga constitutes ink line drawings on paper, while animation is the continuous display of pictures to indicate movement. However, both media use line drawings. The lines drawn by human hands contain temporality and motility through the movement of hands. During the early film era of the 1890s and 1910s, there existed a mode of performance known as lightning sketch, in which an artist would draw a picture in front of an audience and show how the picture gradually changed ( Klein, 1993, 23). This mode of performance embodies the embeddedness of kineticism and temporality in the experience of viewing line drawings. One of Edison’s early films including elements of the lightning sketch, *the Enchanted Drawing* (1900), embodies the media continuum between still line drawings and early animation. The first half shows an unedited shot of an artist drawing a bald, middle-aged man, a wine bottle, and a glass on a canvas placed in front of the camera. In the second half, the camera’s position does not move, but cut editing is used to express a trick effect. The artist takes the drawn wine bottle and glass as real objects and gives the wine to the drawn image of the middle-aged man, causing his expression to change to laughter. *The Enchanted Drawing* reveals the kinetic qualities of line drawings, regardless of media and the spectators’ ability to perceive them.

**Figure 3.**
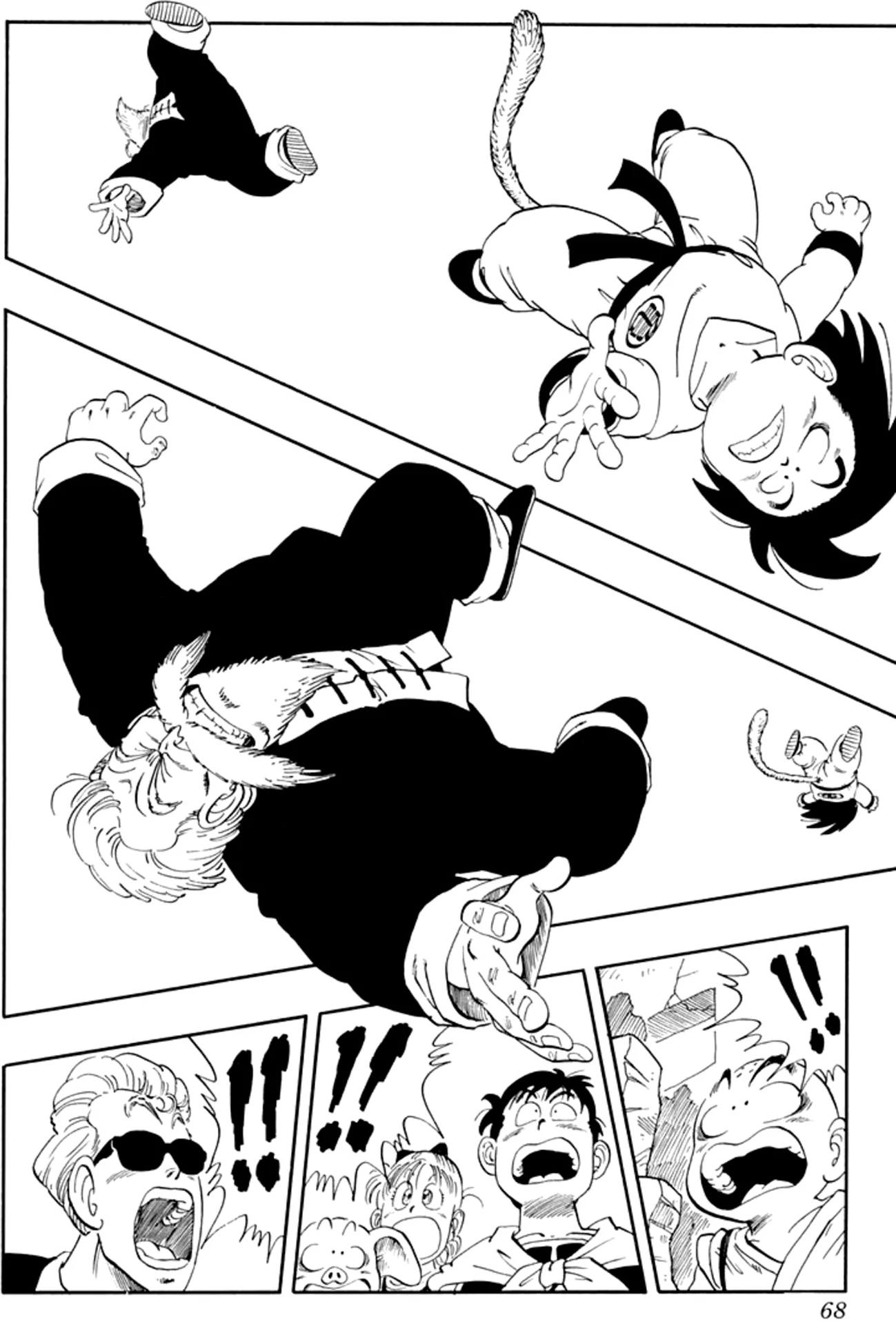
Goku and Jackie Chun’s Cross-Counter (Chapter 53).

The importance of such kineticism gradually increased throughout Toriyama’s career, from *Dr. Slump* (1980-1984) to *Dragon Ball.* From the beginning of *Dr. Slump*, Toriyama’s drawings have been an excellent balance of meticulously drawn machines, such as motorcycles and robots, and pop-deformed humans, which was exceptional by the standard of Japanese manga in the early 1980s. ^4^ However, unlike *Dragon Ball*, *Dr. Slump* does not represent the relative positions of several figures in the three-dimensional space and the kinetic dynamism between them. The latter half of *Dragon Ball*, which shifted to the battle genre depicting the physical confrontation of characters, does not bother with where the battle occurs. The battles take place in an abstract, featureless space, such as some wilderness and the Chamber of Spirit and Time. This can be seen in the fight between Goku and Jackie Chun, a disguise of Kame-Sen’nin, in the early episodes ( Figure 3). While omitting specific descriptions of a three-dimensional space, the relative positions of the two fighting figures are expressed, emphasizing the temporality that flows between the two characters as they fall after the cross-counter.

Rather than discussing the story of *Dragon Ball*, this paper analyzes it as line-drawing images in which kineticism is embedded. In this regard, Susan Napier’s *Anime from Akira to Princess Mononoke: Experiencing Contemporary Japanese Animation* (2001) and its updated edition, *Anime from Akira to Howl’s Moving Castle* (2005) are important studies discussing metamorphosis in Japanese anime. The books are intended as a systematic approach to the various Japanese animations of the time, using Napier’s three modes — “apocalypse,” “festival,” and “elegy” — as a rough classification framework ( Napier, 2005, 12-13). Across these modes, Napier discusses metamorphosis in animation. Based on Paul Wells’ *Understanding Animation*, Napier emphasizes the importance of metamorphosis in animation as follows:

[A]nimation’s emphasis on metamorphosis can be seen as the ideal artistic vehicle for expressing the postmodern obsession with fluctuating identity. What animation scholar Paul Wells describes as “the primacy of the image and its ability to *metamorphose* into a completely different image” is a function of animation that has powerful resonances with contemporary society and culture ( Napier, 2005, 12). [emphasis by the author]

Napier’s argument connects the ability of animation to metamorphose with changing identities in postmodern Japanese society at the turn of the century. Therefore, she discusses metamorphoses in Japanese animation films and TV programs, such as Tetsuo’s collapsing body in *Akira* (Ōtomo Katsuhiro, 1988), the protagonist’s transformation to battle form in *Cutie Honey* (1973-74), the sex/gender switching in *Ranma 1/2* (1989-1992), the tentacled monster in pornographic animation, and the shape-shifting entities in Miyazaki Hayao’s works. Vivian Sobchack (2008, 251-265) and Scott Bukatman (2012) started their arguments with metamorphoses or the free movement of lines, making Napier’s argument common in the English-speaking world.

Napier’s argument inevitably connects with the discussion of “plasmaticity” in animation. The word “plasmaticity” was proposed by the Soviet film director and theorist Sergei Eisenstein. He referred to the plastic transformation of Disney film characters of the 1920s into indefinite shapes as “plasmatic.” Eisenstein appreciated the free-kinetic nature of animation, such as Mickey Mouse piloting the steamboat without permission, being chastised by Captain Pete, and having his torso stretch and contract in ways that would be impossible if he were a human being in *Steamboat Willie* (1928), the world’s first talkie animation. Although plasmaticity has been considered a medium specificity of animation, Eisenstein originally explained it by referring to manga-like line-drawn illustrations. He listed illustrations accompanying *Alice in Wonderland* and woodblock prints depicting Japanese yokai — supernatural beings in Japanese folklore with necks and noses that stretch and contract — as examples of unexpected character deformation ( Eisenstein, 2017, 18-19).

Unlike Eisenstein, Napier does not simply celebrate the possibility of the transformation as an expression of freedom because for her metamorphosis is deeply related to themes, such as sex, sexuality, and childbirth. In *Ranma 1/2*, the protagonist metamorphoses between male and female; however, the overall story follows the order of the male-female binary. In pornographic anime, metamorphosis is often shown in tentacles sex linked to female ecstasy. After pointing out the connection between metamorphosis and sex and sexuality, Napier discusses “abjection” in Tetsuo’s collapsing body in *Akira* based on Julia Kristeva’s argument. Napier describes Tetsuo’s transformation as a grotesque birth scene, as follows:

Tetsuo’s transformations can be viewed as a particularly gruesome form of combined primal and birth scenes: The phallic tentacular arm that expands and contracts ultimately seems to lose itself into an oozing feminine pinkness, which in turn becomes a gigantic baby. This horrifying “birth scene” echoes cinema theorist Barbara Creed’s statement that the act of birth is seen as grotesque “because the body’s surface is no longer closed, smooth, and intact, rather it looks as if it may tear apart, open out, reveal its innermost depths ( Napier, 2005, 45).”

Compared to this description of Tetsuo’s transformation by Napier, Cell’s metaphorical birth scene is not grotesque or horrific. Cell seems to easily create his Juniors without the slightest twinge of pain or significant identity change. This paper argues that the ease, paradoxically, exemplifies that the issues discussed by Napier are concealed in *Dragon Ball*, where the characters are metamorphosed and their sexuality is obscured.

Sexuality, specifically, sexual interest in the female body, is in fact expressed in the adventure part of *Dragon Ball.* In the first volume, Bulma shows substantial nudity. Goku, who had never met a woman before, particularly notices the lack of a penis by tapping the crotch of the female characters. When Goku and Bulma encamp at the very beginning of the story, Goku discovers that Bulma has no penis, which astonishes him more than when fighting a strong enemy. (Toriyama, Ch. 2). Yumcha, Goku’s rival, a desert bandit in the early stages of the story, has a neurotic reaction whenever he sees Bulma because he is unaccustomed to communicating with women (Toriyama, Ch. 8). Yumcha joins Goku and his friends on their journey to find the Dragon Balls to fulfill his wish to become accustomed to women. The other major early characters, such as pig human Oolong and Goku’s master Kame-Sen’nin, are portrayed as lustful individuals, willing to stare at women’s naked bodies (Toriyama, Chs. 4, 5, 12). The following is an important plot point: when all the Dragon Balls are collected to summon Shen Long, the magical dragon that grants wishes, Oolong cries, “Give me a gal’s panties,” thwarting the villains’ ambitions for world domination (Toriyama, Ch.20). Thus, when *Dragon Ball* was an adventure rather than a fighting manga, several references to female bodies as objects of sexual desire were present. ^5^

However, in the latter half of the series, the focus shifts to muscular men’s battles, and the issue of sexuality remains untouched. In *Dragon Ball*, the main characters become couples and have children without depicting romance. Perhaps conforming to the norms of shōnen (boy) manga, the main character, Son Goku, is too ignorant about sex. In this manga world, reproduction takes place by replicating characters without depicting their romance, sexuality, and childbirth. Therefore, in discussing sexuality in *Dragon Ball*, this paper examines the graphic characters that transform their forms and how graphic and biological reproduction are embedded in these characters.

## 3. Results

### 3.1 Plasmatic flesh: Proliferation of temporal and differential change

The characters in *Dragon Ball*, regardless of protagonists or antagonists, transform. The Saiyans, playing an essential role in the story, typically have black hair and eyes. However, when transforming into Super Saiyans, they change to blond hair and blue eyes (Toriyama, Ch. 307). The three major villains who appear in the manga’s latter half transform multiple times. The protagonists always find new battle forms and become stronger to defeat the villains, repeating the basic plot pattern of *Dragon Ball.* The transformation of a character’s appearance is a recurring motif and a significant plot driver in *Dragon Ball.* Therefore, we analyze character transformations in *Dragon Ball* regarding metamorphosis and plasmaticity and argue that Toriyama’s plasmatic motility and fluidity-oriented authorship are embodied by the characters, although this is not always apparent.

Napier’s metamorphosis covers different media-aesthetic qualities because some of the anime, she argues, are based on manga. For example, temporal and differential changes, such as the minute changes in Tetsuo’s arm, are in accordance with the medium specificity of animation as a temporal art form. On the contrary, Ranma’s sex/gender switching when he/she is exposed to water is not depicted with such temporal and differential change. In the original manga of *Ranma 1/2*, Ranma’s change occurs momentarily between one panel and the next. The experience of reading manga is temporal, but the medium of manga is not accompanied by temporality. Thus, the metamorphosis in *Ranma 1/2* is different, in terms of media aesthetics, compared with the other cases that Napier discusses.

Contrarily, Eisenstein’s plasmaticity is similar to the temporal and differential changes of Napier’s metamorphosis. In his words, plasmaticity is “the rejection of the constraint of form … freedom from ossification, an ability to take on any form dynamically ( Eisenstein, 2017, 101).” In other words, plasmaticity, ideally, is not bound to a particular form. Thus, Eisenstein cites the flame as an example of plasmaticity because it is constantly changing. However, the Disney works Eisenstein refers to are tied to specific forms: in *Marbabies* (1938), an octopus arranges its tentacles to look like an elephant. Unlike Ranma’s instant and complete change, the octopus has not turned into an elephant. The octopus remains an octopus as a substance, but by changing the shape of its tentacles, it metamorphically appears to the audience as an elephant. Therefore, as Doi Nobuaki argues, providing animation with a doubled perception in the viewer’s consciousness is important ( Doi, 2009, 57-110). According to Eisenstein and Doi’s arguments, plasmaticity is defined as the status of the viewer’s perception in which expression in animation is not fixed in the particular form represented by the line but is possibly open to another state. The idea of plasmaticity is not necessarily about what is specifically depicted by the ink. However, as Eisenstein cites Disney animated characters from the 1920s, his plasmaticity is almost the same as the temporal and differential changes in Napier’s metamorphosis.

In short, when we reclassify transformation into two categories: 1) temporal differential change, easy to express in temporal art; and 2) instantaneous complete change, expressed in art form without temporality, we can see both types in the manga *Dragon Ball.*

In *Dragon Ball*, as a non-temporal art of manga, instantaneous and complete transformation is found in many places. Characters, such as Oolong and Pu’er, can transform into various creatures and inanimate objects as the story demands. With the Hoi-Poi capsule, structures such as vehicles and dwellings are transformed into small, portable capsules. Further, the main characters transform repeatedly to empower themselves. Saiyans transform into a big monkey form when they see the full moon (Toriyama, Chs. 21, 208, 233, 240). Super Saiyans transform into different fighting forms, such as Super Saiyan 2 and 3, with extended muscles and hair. In addition, all three villains in the work’s second half — Frieza, Cell, and Majin Buu — transform multiple times. Frieza changes his form thrice using his power. Cell transforms from its insect-like shape by absorbing the energy of living organisms through needles in its buttocks. Majin Buu, who is born with a fat body, gets fitter by absorbing others.

Characters not only transform but also fuse with each other. Piccolo is originally an amnesiac Namekian who evacuated to Earth due to famine. After learning that the Namekians have mysterious powers, Piccolo fuses with a Namekian warrior on the planet Namek and gains significant power-ups (Toriyama, Ch. 295). Later, he regains his power by merging with the God of the Earth and returning to his original self (Toriyama, Ch. 360). In the very last stage of the story, the characters merge, even if they are not Namekians. Goku learned a technique called “fusion” from the Metamorans, which powers up characters of equal strength by fusing them. Goten and Trunks, Shin and Kibito, and Goku and Vegeta fuse to become new characters to counter Majin Buu (Toriyama, Chs. 480, 501, 503). These examples embody the instantaneous and complete change in character-focused Japanese manga culture.

**Figure 4.**
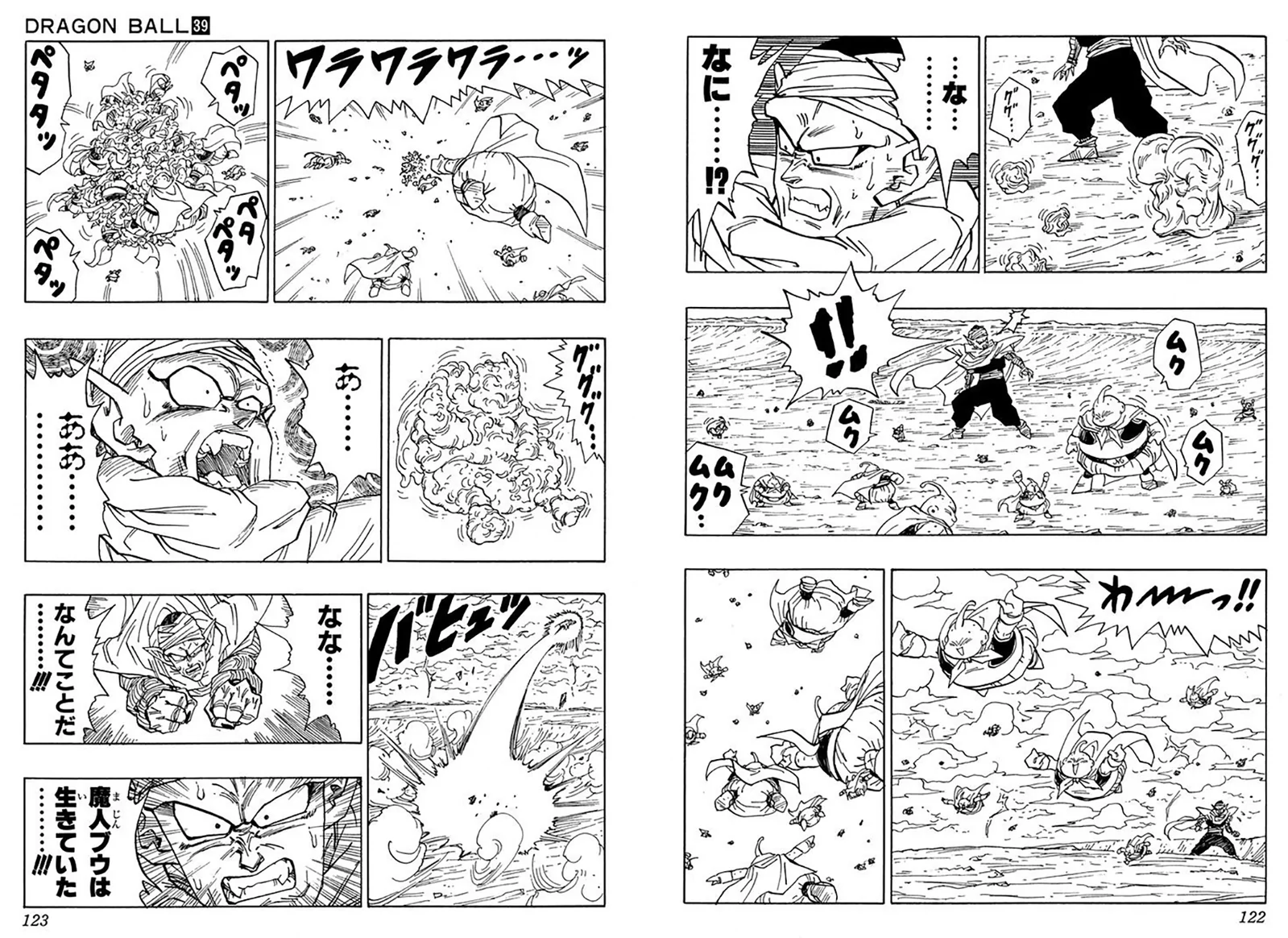
Majin Buu’s Resurrection from Small Pieces (Chapter 468).

At some moments, the transformation or fusion breaks down the character’s outline and exposes the temporal and differential moments. The most prominent example is Majin Buu, who becomes the last villain in the main story of the original manga. Toriyama was tired of drawing battles with strong villains in *Dragon Ball* when he was working on the Buu arc. Majin Buu does not meet the reader’s expectations of a muscular, strong opponent and defies such expectations to allow Toriyama to experiment with the pleasures of free-line movement. Majin Buu is born from smoke and has a non-masculine body in the first form, which can separate and transform at will. Even when Majin Buu is shredded into fine pieces, the pieces transform into smaller Majin Buus, and they merge and resurrect in their original form ( Figure 4) (Toriyama, Ch. 468). In other words, Majin Buu’s shape-shifting flesh embodies the temporal and differential qualities of metamorphosis and plasmaticity, which can loosen the boundaries of character.

Both types of transformations appear in the every corner of the manga *Dragon Ball.* If we examine the entire story according to these two categories of transformation, the instantaneous transformation of Oolong, Pu’er, and Hoi-Poi capsule in the early episodes resurfaces as a temporal and differential dynamism of plasmatic flesh in Majin Buu in the final arc. Here, Toriyama’s play with the free movement of the line, which cannot be reduced to elements such as stories or battles, is revealed. Apart from the superficial narrative of the clash of men’s muscles, Toriyama explored the kinetic freedom of line with ink and paper and his play is actualized as a temporal and differential transformation of the characters.

### 3.2 Graphic Line-Drawn Characters as Vessels of Transformations

Eisenstein’s discussion on fluid plasmaticity focused on the concreteness of the Disney characters. This, ironically, means that the plasmatic fluid character requires a fixed basic form. In this sense, Eisenstein’s and Napier’s arguments for exalting indefinite mobility and metamorphosis cannot be divorced entirely from the argument on character images.

Marc Steinberg’s discussion of the transmedia proliferation of Japanese anime characters explain this. He argues that relatively simple line drawings of characters allow for the transmedia diffusion of characters in the early history of Media Mix, a Japanese version of transmedia storytelling ( Steinberg, 2012, 45-64). For example, he has conducted a transmedia analysis of *Astro Boy* (manga, Tezuka Osamu, 1952-68; TV anime series, 1963-66) and highlighted the phenomenon of the distribution and diffusion of *Astro Boy* characters beyond the media boundary. His research revealed that flat characters, drawn as line drawings, can permeate people’s daily lives using complimentary stickers in chocolate candy boxes. Characters carry what Steinberg calls “dynamic immobility (2012, 6),” a static nature that conversely enables transmedia motility, and, in this sense, the characters have ontological properties that confine motility. The line-drawn characters are, thus, a prerequisite of the plasmatic transformation in *Dragon Ball.* Even though the characters’ appearance changes fluidly in *Dragon Ball*, the existence of the original characters is assumed.

Whether it is Napier’s metamorphosis or Eisenstein’s plasmaticity, *Dragon Ball* characters are filled with such fluidity. This study emphasizes that a character is a vessel that contains fluidity at various levels. Both Napier and Eisenstein discuss the transformation through specific characters. Eisenstein devotes ample time to describing flames, but flames do not appear in specific works. Therefore, the characters that host such fluidity, whether metamorphosis or plasmaticity, become important.

Japanese animation and manga studies have discussed the ontological properties of characters depicted in line drawings. The issues of transformation, metamorphosis, and fusion of characters necessarily echo the arguments in these studies. Itō Gō distinguished between *kyara* (proto character), the aspect of human representation in the fictional world of manga as an image drawn with simple lines, and *kyarakutā* (character), the personality behind the *kyara* images ( Itō, 2014, 110-127). Although each image in the individual panels of the manga is distinct, the readers perceive them as a unified personality. In other words, *kyara* refers to individual images in the individual panels of manga, and *kyarakutā* refers to the coherent personality behind these images that readers assume ( Watabe, 2023, 98-99). For example, in *Ranma 1/2*, the male and female Ranma are graphically different, but the readers know that a consistent personality exists behind these different iconographies according to the narrative development. While the *kyarakutā* is important for understanding the story, it is the nature of the *kyara* that supports the transmedia spread of character culture, as pointed out by Steinberg.

### 3.3 Graphical and biological reproduction

By considering the character as a vessel drawn with lines that confine fluid life, the character theory encounters the issue of reproduction. Itō’s discussion indicates that drawing characters in simple lines allows their graphical reproduction in multiple panels and contexts outside the work ( Itō, 2014, 126). The above-mentioned proliferation of Majin Buu symbolically expresses the reproductive nature of *kyara.* In other words, unlike the temporal art of animation, manga cannot express plasmaticity as a fluctuation of indefinite shape like a burning flame. Instead, manga expresses plasmaticity in the form of a numerical proliferation of *kyara*, or static image that confines the fluidity. Enclosing plasmaticity in characters, then, causes the theme of dual reproduction — the reproduction of images and life — in *Dragon Ball*: the medium-ontological phenomenon of the proliferation of line-drawn *kyara* leads to the intra-narrative biological proliferation of *kyarakutā.*

**Figure 5.**
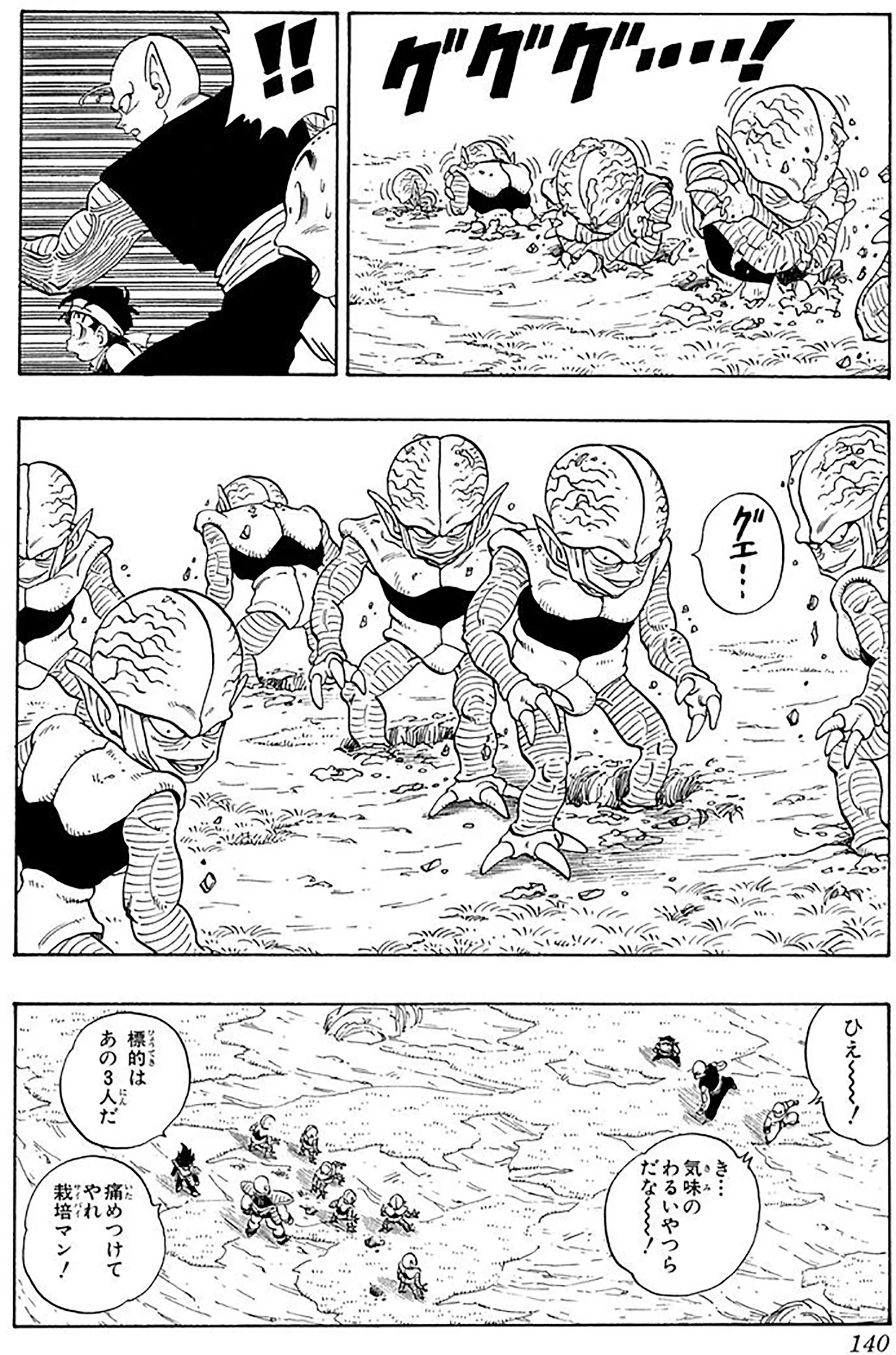
Saibaimen Grow from Seeds Planted in the Ground (Chapter 214).

Double reproduction is evident in the scene germinating Saibaimen. In the Saiyan arc, Goku’s side confronts Vegeta and Nappa, two Saiyans attacking the Earth. Seeing Goku’s side as inferior, Vegeta instructs Saibaimen to fight in his place. Saibaimens, meaning “cultivation man” in Japanese, are improvised combatants that rapidly grow when planted in the ground ( Figure 5). Since Vegeta has the same number of Saibaimen seeds as the number of people on Goku’s side, he uses the remaining seeds to create six graphically identical Saibaimen (Toriyama, Ch. 214). The almost instantaneous process of transformation from seed to humanoid embodies plasmatic change. However, such an explosive transformation of matter is fixed in multiple graphically indistinguishable characters.

Several similar graphically identical individuals are depicted on other occasions. In the Majin Buu arc, Buu can split its irregularly shaped body into multiple graphically identical smaller bodies (see Figure 4). Similarly, Gotenks — the fused form of Goten and Trunks — uses the Ghost Kamikaze Attack technique to create 10 self-explosive ghosts with the same Gotenks head on an irregularly shaped body (Toriyama, Ch. 491). These graphically identical duplicates are also found in the Tien Shinhan, who splits into four bodies, and seven Cell Juniors. Cell’s “delivery” explicitly connects the graphic reproduction of line-drawn images and reproduction as an organism.

Piccolo also expresses dual reproduction. After being beaten by Goku, King Piccolo used all his remaining power to spit an egg (Toriyama, Ch. 161). The egg produced a juvenile that resembled the Demon King, who later took the pseudo-name Majunia when participating in the World Martial Arts Tournament (Toriyama, Ch. 167). After the tournament, Goku and his fellows simply called him Piccolo. King Piccolo was originally an evil part separated from the God of Earth. However, Piccolo, born from the Demon King, later merged with the God of Earth. Thus, the story logically treats King Piccolo and Piccolo (Majunia) as the same character. Piccolo’s fusion with the God of Earth after fusing with Nail shows that fusion does not change Piccolo’s identity at all. The storyline produces multiple Piccolo characters with nearly identical images.

In *Dragon Ball*, the transformation and fusion of characters exemplify Toriyama’s play with free lines. In other words, the kinetic freedom of lines is anchored in the line-drawn characters. The characters drawn in lines are indeed a foundation and a condition that generates fluidity, leading to the graphical reproduction of characters. Characters’ graphical replication in Toriyama’s authorship is, thus, potentially connected to narrative-intrinsic biological reproduction. Here, the issues of fear of reproduction and childbirth that Napier discussed re-emerge.

### 3.4 Abjection of sexuality

Initially, sexuality was frequently specified in *Dragon Ball.* However, as the manga series became increasingly about battles, references to female bodies as objects of sexual desire almost disappeared, and biological reproduction was foregrounded. The main male characters on the protagonist’s side, Goku, Vegeta, and Krillin, married and had children without the depiction of romance, family life, and delivery. Toriyama’s dislike of the romantic comedy genre ( Toriyama, 1984, 130) and the fact that the series was published in an adolescent boy magazine explains the near-complete absence of romance and family life. The proliferation and reproduction of human characters are instead imprinted on various levels in Toriyama’s drawings.

The depiction of Android 18 is a good example of reproduction through a body that lacks sexuality. In the Android arc, Cell is an artificial life made entirely of artificially created organisms, while Androids 17 and 18 are human beings enhanced with artificial organic parts. Toriyama represented Cell and the two Androids as beings of flesh and blood, rather than completely mechanical beings, so that Cell could absorb the two Androids to gain power. Later, Cell ejects Android 18 when Cell is significantly damaged and cannot keep her within him. The ejection of Android 18 is not depicted or foregrounded as a grotesque childbirth in the battle manga context. Such a metaphorical childbirth scene is actualized as a real childbirth lacking specific description in the main plot. Android 18’s reproductive organs and functions, which are unnecessary for fight, mysteriously remain as they are even after she is converted to an Android. She marries Krillin, one of Goku’s companions, and gives birth to a daughter (Toriyama, Ch. 426). A romantic relationship leading up to the birth of the daughter is not depicted at all, as if *Dragon Ball* is trying to “abject” the sexuality and childbirth as a result of romance by making battle the central theme of the manga.

Even heterosexuality, such as references to female bodies as objects of sexual desire, is not represented in *Dragon Ball* after the transition to the battle genre. Instead, biological reproduction is presented, which is cohesive with the existence of Namekians. Piccolo dies in a fight with Vegeta and Nappa, and the Dragon Ball disappears. The God of Earth — the creator of the Dragon Ball — also dies with its counterpart, Piccolo, the evil part of the God of Earth. However, a conversation between Vegeta and Nappa reveals that Piccolo is a Namekian, and the protagonists travel to the Namek planet to resurrect the dead with the power of another Dragon Ball that the Namekians possess. They discover that the Namekians, threatened with extinction, have recovered their population to about 100. The only surviving eldest has given birth to all the Namekians, who live in several settlements around the elders. The word “Namek” is derived from the Japanese word “ *namekuji*,” which means “slug,” and the main Namekian characters, Dende and Nail, are named after “ *denden*mushi,” another Japanese word for slug, and the English word “snail,” respectively. Namekians are hermaphrodites and do not feel love in the manner that humans do.

The “delivery” of Cell is not exceptional in *Dragon Ball* manga. At the beginning of the Buu arc, Android 18 gives birth to a child of Krillin, repeating the motif of childbirth (Toriyama, Ch. 426). Goku and Chichi, and Vegeta and Bulma also have children, but the manga does not depict their romance. In this respect, the Namekians, who reproduce monogenetically, not heterosexually, have ontological characteristics similar to graphically reproducing line-drawn characters. As symbolized in Cell’s “delivery,” symbolic and graphic characters are transformed, fused, and replicated considering plasmatic movement from within. Thus, *Dragon Ball*, except for the initial adventure part, presents graphic and biological reproduction without sex.

Kristeva’s abjection that Napier indicates for Japanese animation in the 1990s seems to operate in the work to make the issues more invisible in *Dragon Ball.* Abjection refers to a reaction in which one tries to dismiss the horrifying yet familiar thing approaching one’s body. It is an ambivalent feeling expressing such a state or action, with the nuance of being horrified at the idea of exposing oneself to it. In *Dragon Ball*, Cell’s “delivery” and the multiplication of flesh in the form of Saibaimen are masked in their horror by being framed in the vessel of the character: what is horrific is the unknown powerful enemies, not the reproduction of life. Through the proliferating characters, the readers can appreciate a double reproduction while remaining oblivious to the horror of childbirth. This is exemplified by the fact that the protagonist, Goku, is utterly unaware of sexual issues throughout the story.

This argument recalls that plasmaticity is related to forgetting for Eisenstein. Theodor Adorno and Max Horkheimer believed that Disney films turned people away from real problems in a capitalist society, as evidenced by Donald Duck being sadistically smashed ( Adorno and Horkheimer, 2002, 110-111). Contrarily, Eisenstein believed that the audience of Disney films would obliterate the capitalist social system but provide a fleeting release ( Eisenstein, 2017, 19). Oblivion is the opportunity to forget the world’s de facto order and be open to the possibilities of an alternative. The fluidity and forgetfulness in Disney films acclimated people to capitalism, as per Adorno and Horkheimer, and were a brief release from capitalism for Eisenstein. Discussing characters in Japanese anime and manga studies within this context can allow us to reinterpret the issues of fluidity and forgetting in *Dragon Ball* as sexuality. In the manga, the free line proliferates and has a fluid presence as plasmatic flesh. However, this plasmatic flesh is to be actualized on the vessel of the characters to replicate the characters as icons. The reproduction of the characters is expressed as devoid of concrete rawness related to sex, sexuality, and childbirth. Thus, in the sexually oblivious world of *Dragon Ball*, a masculine struggle between men is depicted on the surface, with a fluid world of endless dual reproduction behind it.

### 3.5 Light of Annihilation in Japanese Popular Culture

The *Dragon Ball* world is full of elastic fluidity as life. Considering reproduction as a matter of life, there is practically no death because the power of the Dragon Balls can resurrect the dead. The dead characters have angel rings and are trained in martial arts under supernatural beings in the afterlife. They even appear in the real world marked with angel rings for the story’s development. The characters do not consider death seriously. Goku and other characters repeatedly say, “They will come back to life because we have Dragon Balls (Toriyama, Ch. 473).” *Dragon Ball* modifies the graphic status of the characters by displaying or erasing the angelic rings. In the world of *Dragon Ball*, where death does not exist, fluid lines incarnate as the plasmatic flesh of the characters and continue to reproduce themselves.

Consequently, is there no death in this world of *Dragon Ball*? Ōtsuka Eiji argues that manga characters, being mere line drawings, are theoretically invulnerable, similar to Mickey Mouse with a flexible torso in *Steamboat Willie.* According to Ōtsuka, Tezuka Osamu’s depiction of a character who was shot and injured by machin gun fire in his pre-debut study, “Until the Day of Victory (Shōri no Hi Made)” introduced the idea of vulnerable characters into manga ( Ōtsuka, 2009, 133-145). Ōtsuka introduces the realism of the character’s vulnerability as the ethical starting point of postwar manga. Following Ōtsuka’s perspective, the issue of character immortality in *Dragon Ball* can be tied to Toriyama’s preference for American culture. *Dr. Slump*, Toriyama’s other manga, which gained popularity before *Dragon Ball*, refers to a lot of American popular culture. The proliferation of lines with motility in *Dragon Ball* appears to be an obliteration of death in Americanized postwar Japan.

Catherine Malabou’s discussion of “plasticity” provides insight into the issue of lack of death in *Dragon Ball* and, by extension, Ōtsuka’s aforementioned argument. The French philosopher used the concept of “plasticity” to reinterpret Hegel’s dialectic. In Hegel, as read by Kojève and Heidegger, temporality is nullified, and the future is absent. However, by introducing the concept of plasticity, which can both gift and receive forms, it re-finds the self as open to the contingency of the future ( Malabou, 2005, 5-20).

What Malabou refers to here as “plasticity” is different from Eisenstein’s plasmaticity. Plasticity embraces the irreversibility of change and is essentially different from plasmaticity, the idea of not being fixed in a particular form. Malabou applies the concept of plasticity to analyze cinematic and literary works that exemplify this. For instance, Marguerite Duras who “was young for only a very short time ( Malabou, 2012, 56)” and finds herself decisively transformed in her autobiographical novel, *The Lover* (1984), and the female protagonist in Alain Resnais’ *Hiroshima Mon Amour* (1959), who has been thrown out of her hometown because of her romance with a Nazi officer during WWII, revive their memories and start a new life. Thus, the plasticity of Malabou is about the decisive and irreversible transformation of human beings and is not directly related to the free dynamism of line drawings in animation.

Malabou’s concept of “plasticity” is applicable to analyzing manga when she finds a third meaning in the idea: “form blasting,” which is different to the gifting and acceptance of the form. With the invention of the plastic bomb in the 20 ^th^ century, the word “plasticity” was given a third meaning — the instantaneous destruction or annihilation of forms ( Malabou, 2009, 70-77). The meaning of “plasticity” as a destruction of form is quite different from the ability of the medium of plastic art to receive form, such as viscosity or marble, or the ability to provide form, such as education or biological adaptation. In this third sense, the form is destroyed instantly, causing explosive change.

**Figure 6.**
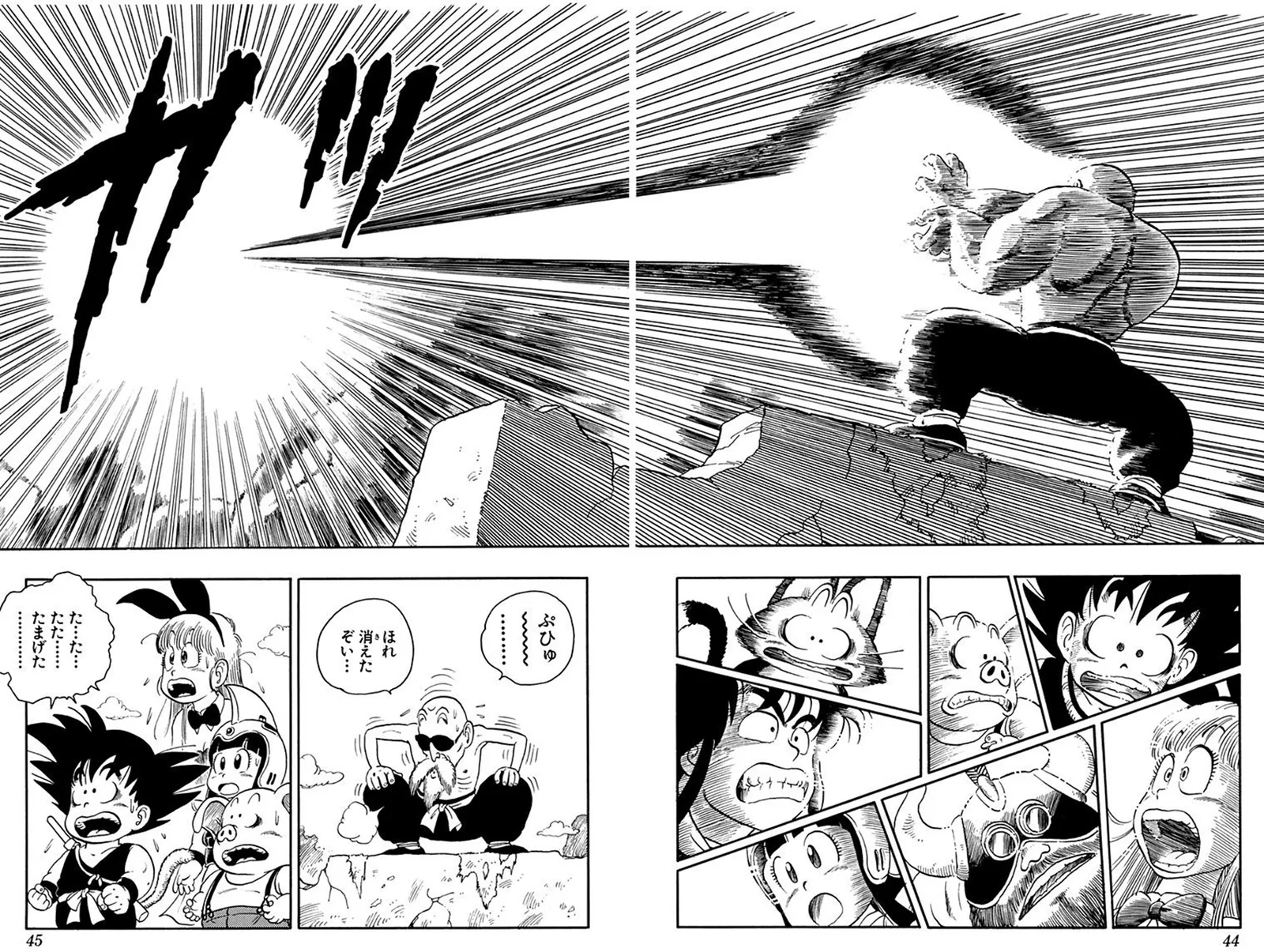
Kame-Sen’nin’s *Kamehameha* (Chapter 14).

This third sense of plasticity is expressed in *Dragon Ball* in the form of the energy waves emitted by the characters, as represented by Kamehameha. In the early story, Goku’s master, Kame-Sen’nin, uses a *Kamehameha*, which releases latent energy from both hands, to extinguish the flames — the very object that Eisenstein considered to be plasmatic —burning the castle of the Ox King ( Figure 6) (Toriyama, Ch. 14). Following the same, the characters in the film perform techniques that emit various energy waves to annihilate their enemies. The characters engage in physical combat and attack by releasing energy waves, mainly from their hands. This energy wave annihilates the three main villains. For instance, Cell and Buu embody plasmatic motility to revive themselves from a single cell and cannot be destroyed in hand-to-hand combat. Goku’s son Gohan releases the *Kamehameha* that extinguishes Cell to the last piece (Toriyama, Ch. 416). In the case of Buu, Goku releases *Genkidama*, which destroys Buu entirely (Toriyama, Ch. 516). Regardless of the intra-narrative life and death of the characters, this light of annihilation can destroy the plasmatic movement and proliferation of cells.

Light poses a real crisis for the characters. The glowing light of the annihilating energy wave is technically an absence of ink on paper. Thus, the lack of ink imparts death to the characters, which graphically proliferate with the plasmatic flesh movement, exemplifying the medium specificity of manga as ink on paper.

The pure white glow is the counterpart of light when considering animation as a medium of light, which is analogous to live-action film. The unfolding of the characters’ deaths by light positions *Dragon Ball* in the genealogy of Japanese popular culture. Several examples in Japanese popular animation series show that light not only deletes an organism but also causes its ontological annihilation. For instance, the great explosion of Neo-Tokyo caused by the psychic Akira’s uncontrolled abilities in *Akira*, the vaporization of human bodies by beam rifles, mega-particle cannons, and nuclear explosions in the *Gundam* series (1979-), and the heat and destructive rays spewed by giant monsters in the Godzilla series (1954-) utilize light as a means to give characters death. Many post-1980 celluloid (cel) anime, like *Dragon Ball* and *Dragon Ball Z*, utilized “transmitted light ( *tōkakō*).” When creators sought to create a striking light image, they started by producing cel images with certain masked areas. These masked cels were then exposed to a strong backlight. By multiple-exposing the strong light image with normally filmed animated images, they created anime that had a brighter glow than those using paint to express light ( Goshima, 2017). In this sense, celluloid anime practically used light to bring annihilation to the medium of anime.

These scenes of catastrophic destruction are inevitably associated with the atomic bomb explosion in Japanese cultural history. While discussing the “invisible light” throughout the modern history of Japanese culture, Akira Lippit discusses the atomic bomb as a “violent photography directly onto the surfaces of the human body” by stating the following:

The catastrophic flashes followed by a dense darkness transformed Hiroshima and Nagasaki into photographic laboratories, leaving countless traces of photographic and skiagraphic imprints on the landscape, on organic and nonorganic bodies alike. The world a camera, everything in it photographed. Total visibility for an instant and in an instant everything rendered photographic, ecstatic, to use Willem de Kooning’s expression, inside out. The grotesque shadows and stains — graphic effects of the lacerating heat and penetrating light — the only remnants of a virtual annihilation. ( Lippit, 2005, 109)

Lippit considers the traces of organic material baked by the light and heat of the atomic bomb as the photographic effect. His argument parallels the characteristics of *Dragon Ball* that this paper has discussed so far. The movement of the lines drawn by the ink gains characters as vessels and multiplies, but the light expressed by the lack of ink demolishes them. The light that causes immense destruction obliterates the problem of flesh, which is forgotten in Japanese popular culture, just as heterosexual reproduction and childbirth are concealed in *Dragon Ball.*

**Figure 7.**
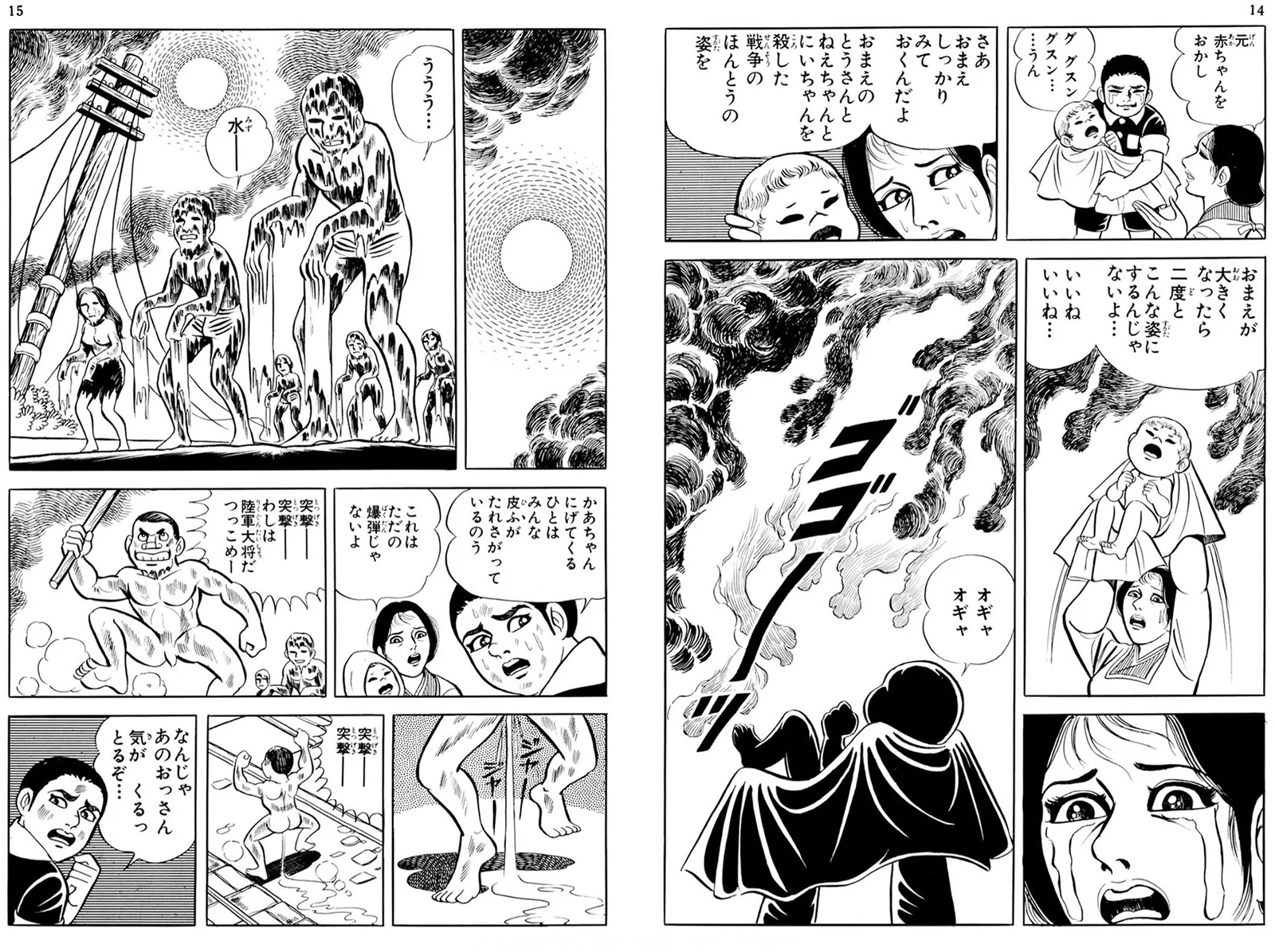
Childbirth on the Day of Atomic Explosion and Melting Bodies of Victims (Nakazawa Kenji, *Barefoot Gen*, vol. 2, 14-15).

By comparing this understanding with Ōtsuka’s ethical critical position already discussed, *Dragon Ball* can be seen as one of the turning points in postwar Japanese manga culture. From the perspective of Ōtsuka, who begins his historical account of postwar Japanese manga, “vulnerable body” inescapably leads to the damage caused by the atomic bomb in the Japanese context. Hence, the manga *Barefoot Gen* (Nakazawa Keiji, 1973-87), which depicted many victims of the atomic bomb explosion over Hiroshima whose skin does not regenerate and whose bodies melt down due to radiation, would be the ideal postwar Japanese manga. In *Barefoot Gen*, Gen’s mother gives birth to Gen’s younger sister on the day the atomic bomb was dropped. Under the light of the annihilation of the atomic bomb explosion, the birth of new life in front of the plasmatic burning flame is placed in parallel with the victims whose bodies are being disintegrated by the radiation with skin hanging off hands and back ( Figure 7). In contrast to Otsuka’s ethical view emphasizing realism, *Dragon Ball* presents a symbolic manipulation of bodies that mask and forget the raw vividness with the aesthetics of the medium of ink line drawing.

## 4. Conclusion

This study places *Dragon Ball* within a theoretical trend in anime and manga studies that emphasizes and analyzes the kinetic nature of the line. Drawing on the work of Susan Napier and Sergei Eisenstein, the study discusses the presence of a tendency toward a certain extent of freedom of line motility in *Dragon Ball.* However, the mobility shown in the manga does not always open the audience’s perception to the possibility of other forms, as Eisenstein argues, but rather represents limited freedom within the confines of the graphic characters. However, being trapped in this frame of character creates a double reproducibility in *Dragon Ball.* The motility of the line is absorbed into the framework of the characters and aims to multiply. In the world of *Dragon Ball*, which avoids facing up to sex and sexuality, the proliferation of lines is represented as graphic reproductions of characters. Therefore, the sexual elements that Napier emphasized are suppressed and obscured in *Dragon Ball.* This study contends that through this double reproducibility, characters multiply to the extent that the death of characters is nullified. With energy waves, like *Kamehameha*, represented by the lack of ink, protagonists can kill the major villains who have plasmatic bodies that can be resurrected even from a single cell. Only the light produced by this lack of ink can annihilate the characters. The annihilation of prolific characters through light replicates the image of the atomic bomb explosion in Japanese popular culture. The argument of this paper facilitates our understanding of the representations of disaster and destruction in various manga and anime.

## Data Availability

No data are associated with this article.

## Footnotes

^1^
Inexpensive figures have been available on *gachapon* vending machines in Japan since the 1980s when the manga was serialized in the weekly magazine. Presently, the franchise offers expensive, realistic figures for adults through a sales promotion strategy called *Ichiban Kuji.*

^2^
For example, Ivan Rakitić, a professional footballer, posted his photo with a figure of Goku when he visited Japan on Instagram on December 18, 2015. https://www.instagram.com/p/_bvn1HSGM6/. For other examples of celebrity fans, see: The Know, “Celebs, athletes give ‘Dragon Ball’ pop culture super status,” *The Denver Post*, January 16, 2019. https://www.denverpost.com/2019/01/16/celebs-athletes-give-dragon-ball-pop-culture-super-status/

^3^
When noting the source of an event in the *Dragon Ball* story, the manga chapter number was considered. This is because the chapters in each volume differ in Japan between the 42-volume manga in the standard edition and the 34-volume complete edition. In the English-speaking world, the first 16 volumes of the 42-volume Japanese series have been published in book form as *Dragon Ball*, and the latter 26 volumes as *Dragon Ball Z.* To avoid confusion caused by such differences between versions, we have decided to indicate chapter numbers.

^4^
In a manga published in 1982-84 that teaches how to draw manga, Toriyama introduces Tezuka Osamu’s manga semiotics, in which characters are created by combining symbols ( Toriyama and Sakuma, 1985). He refers to perspective and motility; however, the content is relatively short.

^5^
References to sex and sexuality caused a controversy in the United States although the original manga series was published in a magazine targeting pre-teen to teenage boys in Japan (“Viz explains,” 2000; “ Maryland School,” 2009).
