# Supplementary material for: Crush of Plasmatic Flesh: Dual Reproduction of Characters and Abjection in Manga Dragon Ball
Source: F1000Res. 2026 Jun 15;14:236. Originally published 2025 Feb 26. [Version 2] doi: 10.12688/f1000research.159864.2 (PMC13550903; doi:10.12688/f1000research.159864.2)
Supplement: Peer review history

# Reviewer report: Reviewer response for version 2

**DOI:** 10.5256/f1000research.198823.r493847

**Published:** 2026-07-09

**Authors:** Nor Afian Yusof

**Affiliations:** 1 Universiti Kebangsaan Malaysia, Bangi, Malaysia

I thank the author for the detailed response to the initial review comments. However, having re-examined version 2 of the manuscript, I find that several key issues remain substantively unaddressed in the text itself, not withstanding the clarifications offered in the author's response letter.

**1. Scope regarding empirical/reception data**

 I accept the author's clarification that this study is designed as a medium-specific formal analysis rather than a reception study, and that this is a legitimate methodological justification. However, I would ask the author to reconsider certain claims in the Abstract and Conclusion that gesture toward broader collective-cultural significance (for instance, the connection drawn to atomic bomb trauma and "post-war Japan"), as such claims implicitly require reception-based or historical support that falls outside the scope the author has explicitly delineated. I recommend that this language be softened accordingly (e.g., "this analysis suggests a possible resonance with..." rather than "offer rich insights into... post-war Japan") to remain consistent with the formal-analytic scope the author has defined.

**2. Comparative analysis**

 The treatment of Akira in the manuscript functions primarily as a theoretical foundation (via Napier's framework) rather than as an analytical comparison conducted on equal footing with Dragon Ball. The additional sentence in the conclusion ("methodology... transferable to other manga/anime") remains a generic assertion without substantive support. I recommend that the author incorporate at least one concrete comparative paragraph, for example, explaining how the mechanics of abjection in Evangelion or Parasyte operate differently from (or similarly to) the dual-reproduction dynamic argued for Dragon Ball in order to substantiate the transferability claim within the text itself, rather than as rhetorical assertion alone.

**3. Close visual reading**

 The addition of a single further chapter reference in Section 3.3 does not fulfill my original request for formal close reading of linework, negative space, and panel composition. The discussion accompanying Figures 1–7 remains largely narrative-descriptive (explaining plot events) rather than a formal analysis of the visual elements themselves. Given that the paper's central thesis rests on claims about the "free kinetic movement of lines," I consider this a methodological gap rather than a matter of stylistic preference. I ask the author to add at least one explicit close-reading example for instance, how line direction or thickness in Figure 4 (Majin Buu) produces the "plasmatic" effect described, or how negative/white space in Figure 6 (Kamehameha) visually "erases" the character as concrete evidence supporting the theoretical argument.

**4. Masculinity**

 I accept the author's clarification that masculinity is treated as a surface ideology rather than a neglected topic, and that this is consistent with the paper's thesis. However, the "would duplicate" argument is not fully convincing literature on Japanese hyper-masculinity (e.g., Kinsella, Galbraith) could in fact strengthen rather than duplicate the existing argument, by providing a theoretical framework for how muscular-body imagery functions as an ideological mechanism within Japanese visual culture. I recommend at least one brief reference or paragraph connecting the concept of a "masculine surface concealing an underlying logic" to existing scholarship on masculinity in Japanese popular culture.

**Decision**

 Approved with Reservations (maintained). I recommend conditional acceptance, contingent on the author addressing Points 2 and 3 specifically within the manuscript text itself (not merely in the response letter) prior to indexing, as both involve substantive methodological gaps rather than differences of stylistic approach.

Is the work clearly and accurately presented and does it cite the current literature?

Partly

If applicable, is the statistical analysis and its interpretation appropriate?

Not applicable

Are all the source data underlying the results available to ensure full reproducibility?

Partly

Is the study design appropriate and is the work technically sound?

Partly

Are the conclusions drawn adequately supported by the results?

Partly

Are sufficient details of methods and analysis provided to allow replication by others?

Partly

Reviewer Expertise:

graphic design and animation

I confirm that I have read this submission and believe that I have an appropriate level of expertise to confirm that it is of an acceptable scientific standard, however I have significant reservations, as outlined above.

# Reviewer report: Reviewer response for version 2

**DOI:** 10.5256/f1000research.198823.r495834

**Published:** 2026-07-08

**Authors:** Christopher Smith

**Affiliations:** 1 University of Florida, Gainesville, Florida, USA

The present article is an exciting new contribution to both scholarship on *Dragonball *and to manga theory. It examines the use and absence of line as a semiotic tool that denotes the fluidity and plasticity of bodies, seemingly presented as hard-edged, defined, and masculine, but actually frequently transforming, merging, splitting, and reproducing themselves. Ingeniously, the author points out that light, depicted as the absence of ink, is what destroys these bodies and stops their plasmatic reproduction.

 The article is a welcome contribution to the field, and its analysis is intriguing. I only have a couple of suggestions:

1. The article argues that reproduction happens in *Dragonball *without depiction of sexuality, romance, delivery, or “family life.” (Section 3.4) While the point about lack of sexuality and romance is well taken, in fact the series does depict family life, even if it is peripheral to the battles. This is significant because the family life rendered in *Dragonball* transforms strong female warriors into mothers and housewives; e.g., Chichi transforms from a capable fighter into an “education mama,” Android 18 transforms from a terrifying and powerful villain into a wife and mother who, notably, loses to the pathetic Mr. Satan to support the household finances. In the spinoff material, Videl also becomes devoted to motherhood. In other words, while there might be a lack of sexuality or romance, the gendered family (the social site of reproduction) and its roles for women are still very much present and powerful, which the article might acknowledge.
2. The article mostly treats *Dragonball *as a case study for examining manga line, ink, and the lack thereof. This is a fantastic analysis, but it would be nice if the article could also clearly state, perhaps just in the conclusion, how this analysis challenges or transforms our reading of *Dragonball* itself.

Despite these minor suggestions, the article is analytically rigorous, participates in the ongoing scholarly discourse, and is a welcome and timely contribution to the field.

Is the work clearly and accurately presented and does it cite the current literature?

Yes

If applicable, is the statistical analysis and its interpretation appropriate?

Not applicable

Are all the source data underlying the results available to ensure full reproducibility?

No source data required

Is the study design appropriate and is the work technically sound?

Yes

Are the conclusions drawn adequately supported by the results?

Yes

Are sufficient details of methods and analysis provided to allow replication by others?

Yes

Reviewer Expertise:

Modern Japanese literature, manga, anime, Japanese pop culture, postmodernism.

I confirm that I have read this submission and believe that I have an appropriate level of expertise to confirm that it is of an acceptable scientific standard.

# Reviewer report: Reviewer response for version 2

**DOI:** 10.5256/f1000research.198823.r495833

**Published:** 2026-07-07

**Authors:** Valentina-Andrada Minea

**Affiliations:** 1 University of Munster, Münster, Germany

This article presents an original and theoretically ambitious analysis of *Dragon Ball*, examining the relationship between graphical transformation, biological reproduction, and abjection through the frameworks of Susan Napier, Sergei Eisenstein, and Japanese manga studies. Rather than approaching *Dragon Ball* primarily as a narrative or cultural phenomenon, the author focuses on the medium-specific properties of manga itself, arguing that the fluidity of ink lines and the reproduction of graphical characters form the basis for a distinctive aesthetic of transformation. The article further proposes that this graphical reproduction parallels biological reproduction while simultaneously suppressing explicit representations of sexuality, culminating in an insightful discussion of light, annihilation, and postwar Japanese visual culture.

 One of the major strengths of the paper is its interdisciplinary engagement with animation theory, manga studies, philosophy, and media aesthetics. The author successfully combines established theoretical perspectives with close readings of *Dragon Ball*, producing an interpretation that is both original and intellectually stimulating. The discussion of plasmaticity, kyara theory, and graphical reproduction is particularly compelling, and the article offers a valuable contribution to scholarship on manga and Japanese visual culture.

 The manuscript is also well grounded in the primary source material. Numerous examples from *Dragon Ball* effectively support the theoretical claims, and the revised version has further strengthened these connections through additional references and clarifications. The conclusion clearly articulates the broader applicability of the proposed analytical framework beyond *Dragon Ball*, increasing the paper's relevance for future research in anime and manga studies.

 The manuscript is generally well written and logically structured. The progression from theoretical foundations to textual analysis and finally to broader cultural implications is coherent and easy to follow despite the conceptual complexity of the subject.

 I have only a few minor suggestions that could further strengthen the manuscript:

- Some of the theoretical sections are particularly dense and introduce multiple conceptual frameworks in rapid succession. Adding a few brief transitional sentences summarizing how each framework builds upon the previous discussion would improve readability for readers who are less familiar with anime and manga theory.
- The connection between the concepts of plasmaticity, reproduction, and abjection is convincing, but occasionally the transitions between these concepts could be made slightly more explicit, particularly when moving from medium-specific analysis to broader cultural interpretations.
- The discussion of postwar memory and the atomic bomb imagery is thought-provoking. A brief acknowledgment that this represents one interpretative framework among several possible readings would further reinforce the scholarly balance of the argument.

These suggestions are intended only to improve clarity and accessibility. They do not affect the originality, methodological soundness, or scholarly value of the manuscript.

 Overall, this is a thoughtful, innovative, and well-researched contribution to manga and anime studies. It advances an original theoretical perspective on *Dragon Ball* while engaging critically with existing scholarship. I believe the manuscript is scientifically sound and suitable for indexing after minor revisions addressing the points above.

Is the work clearly and accurately presented and does it cite the current literature?

Yes

If applicable, is the statistical analysis and its interpretation appropriate?

Not applicable

Are all the source data underlying the results available to ensure full reproducibility?

No source data required

Is the study design appropriate and is the work technically sound?

Yes

Are the conclusions drawn adequately supported by the results?

Yes

Are sufficient details of methods and analysis provided to allow replication by others?

Partly

Reviewer Expertise:

Japanese Anime, Religion, Interreligious and Intercultural Communication, Philosophy.

I confirm that I have read this submission and believe that I have an appropriate level of expertise to confirm that it is of an acceptable scientific standard.

# Reviewer report: Reviewer response for version 1

**DOI:** 10.5256/f1000research.175650.r371095

**Published:** 2025-04-16

**Authors:** Nor Afian Yusof

**Affiliations:** 1 Universiti Kebangsaan Malaysia, Bangi, Malaysia

**Overall Assessment**

 This article presents a critical and nuanced analysis of the mechanisms of reproduction and character transformation in Dragon Ball through the lens of animation theory and visual cultural studies. By employing Eisenstein’s concept of plasmaticity, Napier’s metamorphosis, and Kristeva’s theory of abjection, the author successfully frames Dragon Ball as a text that not only depicts physical battles but also explores the tension between fluid identity and absolute destruction.

 The intellectual strength of this paper lies in its ability to connect the aesthetics of manga as a medium with broader cultural and historical dimensions, particularly in referencing post-war Japan and the visual legacy of the atomic bomb. Thus, this article has the potential to become a significant reference in manga and anime studies, as well as in broader discourses on visual culture and media theory.

 However, there are certain methodological and analytical aspects that require further elaboration and justification to elevate the arguments to a higher level of academic rigor.

**Strengths of the Article**

 1. Rich and Interdisciplinary Theoretical Framework

 The author integrates theories from diverse fields—animation studies, semiotics, Japanese cultural studies, and psychoanalytic theory—in a complex yet structured manner. The use of Eisenstein’s plasmaticity and the application of Kristeva’s abjection theory to *Dragon Ball* demonstrate the author’s ability to conduct a deep reading of visual texts.

 2. In-Depth Aesthetic and Narrative Analysis

 This article offers a fresh interpretation of *Dragon Ball*, shifting the focus from conventional readings that view it as an action manga to a conceptual mapping of dual reproduction. By linking character transformations to the fluidity of ink in manga, the author successfully illustrates how the technical aspects of the medium shape cultural and ideological meanings.

 3.Contextualization within Japanese Cultural History

 One of the greatest strengths of this paper is its ability to connect motifs of destruction and light in *Dragon Ball* with Japan’s visual history, particularly in the shadow of post-Hiroshima. The discussion of energy explosions in anime and manga as echoes of wartime tragedies adds a broader dimension to the discourse, making the article more relevant to contemporary Japanese cultural studies.

**Weaknesses and Suggestions for Improvement**

 1. Lack of Empirical Data or Case Studies Supporting Claims.

 While the article is rich in theoretical analysis, some claims regarding audience reception and cultural implications are made without sufficient empirical support. For example, the argument that *Dragon Ball* reflects Japan’s collective fear of fluid identity and post-war destruction requires additional references from audience reception studies or visual cultural history.

 Suggestion **:**

 The author is advised to include reception studies, fan interviews, or at least reference existing research discussing the reception of *Dragon Ball* in Japan and globally to strengthen the validity of their arguments.

 2. Lack of Comparative Analysis with Other Anime and Manga Texts.

 The article discusses *Dragon Ball* in depth but does not sufficiently compare it with other works featuring similar themes of character transformation, such as *Akira*, *Neon Genesis Evangelion*, or *Parasyte*. Such comparisons could provide a more critical perspective on how *Dragon Ball* differs from or aligns with narratives of transformation and destruction in anime culture.

 Suggestion:

 Adding comparisons with other manga or anime that also focus on bodily and identity transformations would reinforce the argument that the phenomena discussed in this article are not isolated but part of a larger trend in Japanese visual culture.

 3. Imbalance Between Visual and Narrative Analysis.

 Although the article emphasizes the importance of manga aesthetics in shaping meaning, discussions on visual aspects are often overshadowed by more abstract theoretical analysis. For instance, references to specific manga panels in *Dragon Ball* are limited, weakening the reader’s ability to directly observe how the described phenomena are translated into the formal aspects of the manga itself.

 Suggestion:

 Incorporating more close readings of specific panels in *Dragon Ball*, with discussions on how linework, negative space, and panel composition contribute to meaning-making, would strengthen the article’s central arguments.

 4. Overemphasis on Sexuality While Neglecting Masculinity Issues.

 The article discusses how *Dragon Ball* erases sexuality and replaces it with asexual and mechanistic reproduction. However, the aspect of masculinity highlighted in the narrative of muscular male fighters is not explored in sufficient depth.

 Suggestion:

 The author could expand the analysis by linking masculinity in *Dragon Ball* to theories of hyper-masculinity in Japanese popular culture, as discussed by scholars such as Sharon Kinsella or Patrick Galbraith. This would create a more balanced analysis, moving beyond the erasure of sexuality to examine how *Dragon Ball* shapes masculine identities in anime culture.

**Conclusion and Peer Review Decision**

 Overall, this article is a valuable contribution to anime and manga studies, offering an innovative perspective on *Dragon Ball* as a complex visual text. The theoretical approach is robust, and the discussion of dual reproduction and the erasure of sexuality is a discourse that deserves further attention in Japanese cultural studies.

 However, certain methodological and analytical aspects could be improved to elevate the article to a higher academic standard:

 - Including empirical data or reception studies to support cultural arguments.

 - Adding comparisons with other manga or anime with similar themes.

 - Strengthening visual analysis with more close readings of manga panels.

 - Expanding the discussion on masculinity to balance the analysis of sexuality.

**Peer Review Decision:** **Accepted with Revisions**

 This paper has a strong foundation and is worthy of publication after addressing the suggested improvements. With reinforced empirical evidence, broader comparative analysis, and more detailed visual references, this article has the potential to become a key reference in anime and manga studies within the context of Japanese visual culture.

 Here are my comments regarding the questions above:

 1. Is the work clearly and accurately presented, and does it cite current literature?

 Answer: Partly

**Comment:** The article is written in a deeply theoretical and philosophical style, but its presentation can sometimes be convoluted and difficult for non-specialists in cultural theory or body philosophy to follow. Key concepts such as "abjection," "dual reproduction," and "plasmaticness" are discussed with references to major figures like Kristeva and Eisenstein, but there is a lack of engagement with contemporary literature—particularly in current manga studies or modern narrative theory. Some references are also rather dated and do not reflect the latest discourse in the field.

 2. Is the study design appropriate, and is the work technically sound?

 Answer **:** Partly

**Comment:** This study is more conceptual than empirical or quantitative, so a discourse analysis/visual criticism approach is appropriate. However, the conceptual framework is not systematically explained, making some arguments appear academically unsound in terms of analytical rigor. The hermeneutic approach to manga like *Dragon Ball* is valid but could be strengthened with a more explicit and consistent theoretical framework.

 3. Are sufficient details of methods and analysis provided to allow replication by others?

 Answer **:** Partly

**Comment:** Since the article is theoretical and discourse-based, it does not involve empirical methods that can be replicated. However, it lacks clear methodological explanations for visual analysis—such as how manga panels were selected, how conceptual categorizations were made, or how the text was semiotically coded. This makes it difficult for other researchers to replicate or expand upon the study.

 4. If applicable, is the statistical analysis and its interpretation appropriate?

 Answer **:** Not applicable

**Comment:** The article does not involve any statistical analysis. It is interpretive and philosophical in nature.

 5. Are all the source data underlying the results available to ensure full reproducibility?

 Answer **:** Partly

**Comment:** The primary data consists of *Dragon Ball* manga material, but the article does not provide sufficient specific panel references or visual citations to support each argument. Some references to visuals or narratives are too general, lacking image support or exact chapter locations, making it difficult for readers to verify the evidence themselves.

 6. Are the conclusions drawn adequately supported by the results?

 Answer **:** Partly

**Comment:** The conclusion that character bodies in *Dragon Ball* serve as sites of abjection and dualistic identity reproduction is intriguing and conceptually supported by theoretical arguments. However, textual and visual evidence remains somewhat limited and does not always fully substantiate the claims made. The article requires more primary data (visual examples, narrative excerpts) to better support its interpretations.

Is the work clearly and accurately presented and does it cite the current literature?

Partly

If applicable, is the statistical analysis and its interpretation appropriate?

Not applicable

Are all the source data underlying the results available to ensure full reproducibility?

Partly

Is the study design appropriate and is the work technically sound?

Partly

Are the conclusions drawn adequately supported by the results?

Partly

Are sufficient details of methods and analysis provided to allow replication by others?

Partly

Reviewer Expertise:

graphic design and animation

I confirm that I have read this submission and believe that I have an appropriate level of expertise to confirm that it is of an acceptable scientific standard, however I have significant reservations, as outlined above.

# Response

**Published:** 2026-04-13

**Authors:** Kohki Watabe

**Affiliations:** University of Tsukuba, Japan

I thank the reviewer for the careful and constructive reading of the manuscript. Below I respond to each of the four points raised.

**1. Lack of empirical data or reception studies**

 The reviewer suggests adding reception studies or fan interviews to support arguments about audience perception and cultural implications. I appreciate this suggestion; however, the present study is intentionally designed as a medium-specific formal analysis of the manga text rather than a reception study. The paper examines the visual and graphical properties of *Dragon Ball* as a media object, drawing on animation theory and manga studies rather than audience ethnography. Incorporating reception data would require a substantially different methodological framework and is beyond the scope of this study. I have clarified the paper's scope in the Methods section to make this boundary more explicit.

**2. Lack of comparative analysis with other anime and manga texts**

 The reviewer suggests broader comparison with works such as *Akira*, *Neon Genesis Evangelion*, and *Parasyte*. I note that *Akira* already plays a significant comparative role in the paper: Tetsuo's transformation in *Akira* is discussed at length in Section 2 as a counterpoint to the abjection dynamics in *Dragon Ball*, and the Neo-Tokyo explosion is referenced in Section 3.4 in the context of light and annihilation in Japanese anime. The paper thus does situate *Dragon Ball* within a broader landscape of Japanese visual culture. A systematic comparison with additional titles would, however, shift the paper's focus away from its core argument about the medium-specific properties of *Dragon Ball* as a manga text. I have added a brief note in the conclusion to clarify how the analytical framework developed here might be extended to other works.

**3. Imbalance between visual and narrative analysis**

 The reviewer suggests more close readings of specific manga panels. I have added a further chapter reference in Section 3.3 to strengthen the grounding of the argument in the primary source material. I would also direct the reviewer's attention to Figures 1–7, each of which is accompanied by detailed analytical discussion in the main text, and to the extensive chapter-specific citations throughout Sections 3.1–3.4. The paper's analytical approach is to treat selected panels as evidence of broader medium-ontological patterns rather than to perform exhaustive panel-by-panel commentary; I believe the existing visual apparatus adequately supports this approach.

**4. Overemphasis on sexuality while neglecting masculinity**

 I respectfully suggest that this comment reflects a misreading of the paper's argument. The paper does not simply emphasize sexuality; rather, its central argument is precisely that *Dragon Ball* systematically *suppresses* sexuality and romance in favor of an asexual, graphical mode of reproduction. The surface narrative of muscular masculine combat is acknowledged—and indeed foregrounded in the Introduction—as the mechanism through which this suppression is effected. In other words, the paper treats masculinity not as an absent topic but as the surface ideology that renders the underlying logic of dual reproduction invisible. Adding a separate analysis of hyper-masculinity along the lines suggested by the reviewer would duplicate rather than complement the paper's existing argument.

# Reviewer report: Reviewer response for version 1

**DOI:** 10.5256/f1000research.175650.r371099

**Published:** 2025-03-25

**Authors:** Laurence Green

**Affiliations:** 1 University of the Arts London, London, UK

While this paper holds an interesting premise, and approach - combining as it does the vastly popular manga Dragonball with a theory of 'line' that builds on previous work by the likes of Napier (as well as chiming neatly with the themes raised in the British Museum exhibition on manga, for example), the end result feels muddled, and overly intellectualized. While the theory may be solid, the delivery is scattershot - continually claiming to avoid narrative and focusing on the line itself, but ultimately spending more time on explaining character elements of the manga, than a convincing unpacking of the drawings themselves.

 There is definite academic merit here with a solid theoretical basis, but in its current state, it is not an enjoyable read - the textual analysis could do with substantial editing to clarify the points it is making, and all in all, aim toward a more direct presentation of its findings. As it is, it just feels like a self-reflective exercise in academic intellectualism. There needs to be much more done to make the analysis convincing - why should we care about all this? There is some hint of this at the end of part 3.4 with the discussion of Eisenstein, but it doesn't go deep enough in specific relation to manga/anime. More recent work by the likes of Dennison, Lamarre etc may help find links between this study and convincing arguments of anime's relevancy as a global media property. The conclusion claims the study forms part of a 'trend' in anime/manga studies, but ultimately relies too heavily on Napier only - the citation base is solid, but could be broadened.

Is the work clearly and accurately presented and does it cite the current literature?

Partly

If applicable, is the statistical analysis and its interpretation appropriate?

Not applicable

Are all the source data underlying the results available to ensure full reproducibility?

No source data required

Is the study design appropriate and is the work technically sound?

Yes

Are the conclusions drawn adequately supported by the results?

Partly

Are sufficient details of methods and analysis provided to allow replication by others?

Yes

Reviewer Expertise:

Japanese Studies / Anime Studies / Film Studies

I confirm that I have read this submission and believe that I have an appropriate level of expertise to confirm that it is of an acceptable scientific standard, however I have significant reservations, as outlined above.

# Response

**Published:** 2026-04-13

**Authors:** Kohki Watabe

**Affiliations:** University of Tsukuba, Japan

I am grateful to the reviewer for such an engaged and perceptive reading of the manuscript. The reviewer's comments have been genuinely helpful in clarifying the paper's argument and improving its presentation. I respond to each point below.

**On the overall presentation and argument**

 The reviewer's observation that the paper feels "muddled" and that it claims to focus on the line while spending more time on character elements is a valuable one, and I understand why the paper can read this way. I have revised the conclusion to make the theoretical logic more explicit: the paper's core claim is that in the static medium of manga, plasmaticity *cannot* be expressed as pure line movement but must pass through the vessel of the character ( *kyara*)—and it is this necessary mediation that generates the "dual reproduction" central to the argument. I hope the revised conclusion makes clearer why the paper moves between line and character, and why this movement is the argument rather than a distraction from it.

**On "why should we care"**

 The reviewer is right to press on this point, and I appreciate the sharpness of the challenge. I have added a passage to the conclusion that more directly articulates the broader stakes of the analysis: the paper's framework for reading how medium-specific properties of ink and line encode cultural and historical meaning offers a transferable methodology for manga and anime studies more broadly, and connects the formal analysis to Japan's postwar visual imagination in ways that I hope will resonate with readers beyond the immediate field.

**On Lamarre and Dennison**

 The reviewer's suggestion to engage with Lamarre and Dennison is well taken and has pushed me to think more carefully about how to position this study within current debates. I have added a footnote in Section 2 that engages with Lamarre's concept of animetism ( *The Anime Machine*, 2009). As the note explains, Lamarre's framework focuses on the moving image and cel animation technology, whereas the present study addresses the static medium of manga—but the footnote acknowledges the productive tension between the two approaches and situates the present study more clearly in relation to this influential body of work. Regarding Dennison's *Anime: A Critical Introduction* (2015), her focus on transnational genre history and industrial circulation is somewhat outside the formal and medium-specific scope of the present paper; I have addressed this point in the response rather than in the text itself, but I am grateful to the reviewer for prompting me to consider how the paper's argument might speak to those broader debates.

**On over-reliance on Napier**

 The reviewer's point about Napier is well taken. Napier is necessarily central to this paper because her account of metamorphosis and abjection in Japanese animation provides the direct theoretical foil for the argument about *Dragon Ball*; without that interlocutor, the paper's contribution would be harder to define. That said, I take the reviewer's point that the conclusion's claim to situate the paper within a broader "trend" in anime/manga studies should be better substantiated. The addition of the Lamarre footnote, together with the existing engagement with Steinberg, Itō, Ōtsuka, Bukatman, and Lippit, I hope, goes some way toward demonstrating that the paper is in dialogue with a wider field. I have also revised the relevant passage in the conclusion to frame the paper's position more carefully.

**On the citation base**

 I am grateful for the reviewer's encouragement to broaden the citation base. The Lamarre footnote represents the most substantive addition in this regard. I believe the existing bibliography—spanning English-language and Japanese-language scholarship across animation studies, manga studies, film theory, and philosophy—reflects the interdisciplinary scope of the argument, and I hope the revised version demonstrates this more effectively.
